# Giant Pelvic Retroperitoneal Epidermoid Cyst: A Rare Case Report

**DOI:** 10.1155/2012/981387

**Published:** 2012-10-22

**Authors:** F. Z. Fdili Alaoui, A. Oussaden, H. Bouguern, H. El Fatemi, M. A. Melhouf, A. Amarti, K. Ait Taleb

**Affiliations:** ^1^Department of Obstetrics and Gynecology II, University Hospital Hassan II, BP1835 Atlass Fez, Morocco; ^2^Visceral Surgery Department, University Hospital Hassan II, BP1835 Atlass Fez, Morocco; ^3^Department of Anatomic Pathology, University Hospital Hassan II, BP1835 Atlass Fez, Morocco

## Abstract

Epidermoid cyst is a frequent benign cutaneous tumor. The pelvic localization does not occur very often. The literature that taps into such cases is very limited in scope. Here is a report of a 27-year-old woman with a giant pelvic retroperitoneal epidermoid cyst. The use of ultrasound exploration and computed tomography has indicated ovarian origins. The surgery also revealed a retroperitoneal epidermoid cyst, uterus and ovaries were all intact. The evacuation of a cyst was found to contain lamellas of keratin. Histology permitted us to confirm the diagnosis. The patient was faring well after two years of followup.

## 1. Introduction 

Epidermoid cyst is a common cutaneous benign tumor developed from ectodermal inclusion. Patients with an age ranging from 19 to 45 are most likely to be contaminated with this tumor [1].

The most common locations of epidermoid cysts are the face followed by the trunk and the neck, in that order [2]. However, some exceptional locations are reported: brain [3], bone [4], testis [5], penis [6], clitoris [7], spleen [8], and kidney [9].

 The pelvic retroperitoneal cysts are rare to come across [10]. We report a case of giant pelvic retroperitoneal epidermoid cyst observed at the Department of Gynecology and Obstetrics II, University Hospital Hassan II, Fez, Morocco.

## 2. Case

A 27-year-old single woman, G0P0, showed the following symptoms: steadily growing swelling in the abdomen for 6 months, compounded by pelvic pain, and coupled with intermittent constipation and dysuria as a sign of compression. 

 The patient had her menarche at the age of 14, and the menstrual cycles were regular without abnormal uterine bleeding. The physical examination revealed the firm, mobile and insensitive abdominal pelvic mass reaching the umbilical point; whereas the rest of examination revealed no abnormalities.

The abdominal ultrasound showed a giant abdomino-pelvic well-circumscribed hypoechogene mass separated from the uterus. The ovaries could not be visualized.

**Figure 1:**
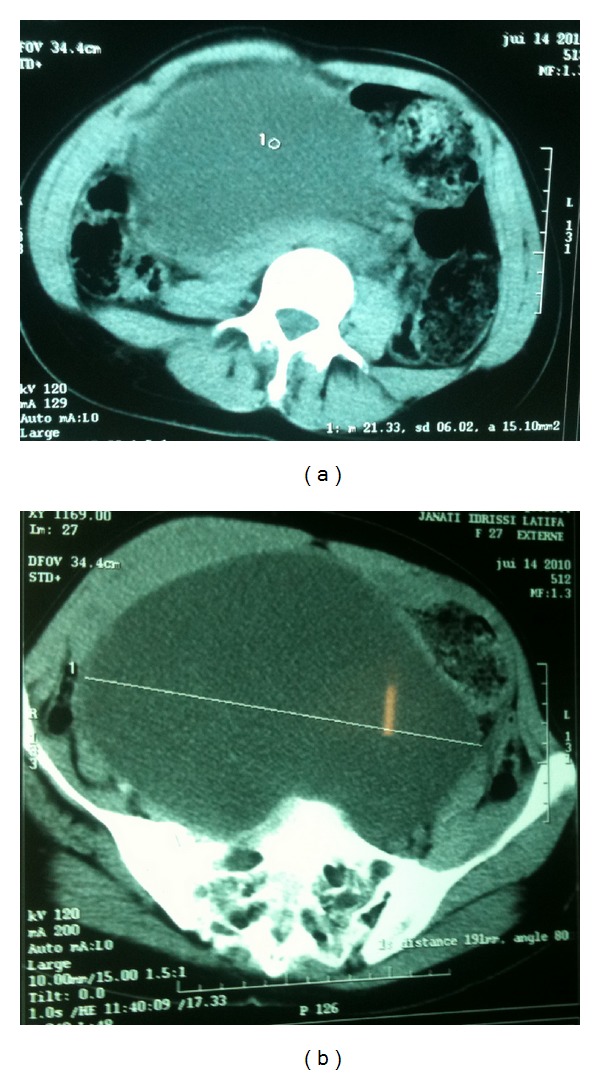
Computed tomography showed a wellcircumscribed mass displacing the uterus forward with left ureterohydronephrosis respecting the cortex suggesting a giant ovarian cyst.

The computed tomography showed a well-circumscribed mass, measuring 20 cm, with fluid density not taking the contrast, without walls or vegetations, displacing the uterus forward with the left ureterohydronephrosis respecting the cortex; the ovaries could not be visualized and the diagnosis pointed to a giant ovarian cyst (Figure 1). Laboratory preoperative investigations were normal.

**Figure 2:**
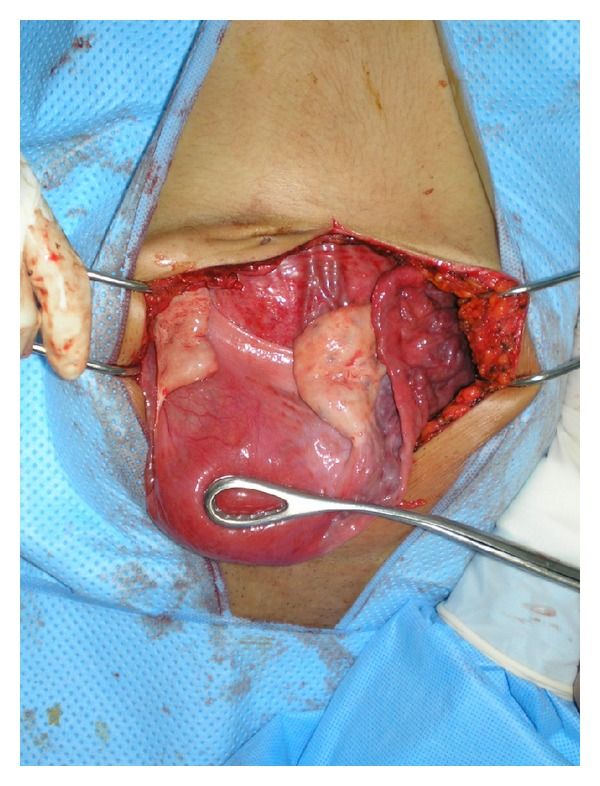
Surgical exploration showed a 25 cm retroperitoneal cyst. Ovaries and uterus were normal.

**Figure 3:**
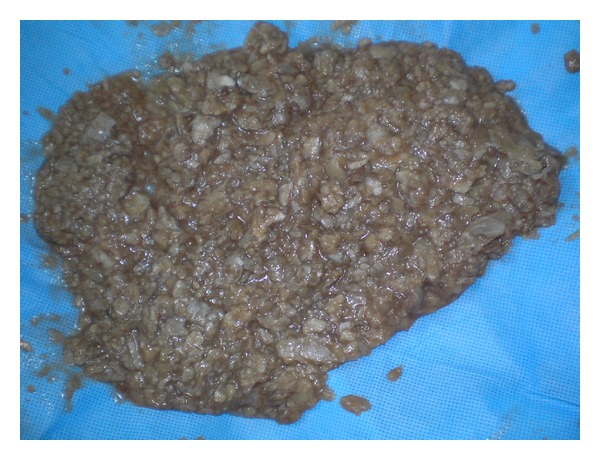
An accidental rupture of the cyst gives way out of the slats reminiscent of keratin.

Median laparotomy was performed and a 25 cm retroperitoneal cyst was found. Ovaries and uterus were normal (Figure 2). After opening the peritoneum (a general surgeon was involved during the operation); we discovered a giant retrorectal firm mass of whitish color, surrounded by pseudocapsule. Under tension, an accidental rupture of the cyst can pour out of the slats, being thus reminiscent of keratin (Figure 3). We performed a complete evacuation of the cyst, and the wall was completely resected.

**Figure 4:**
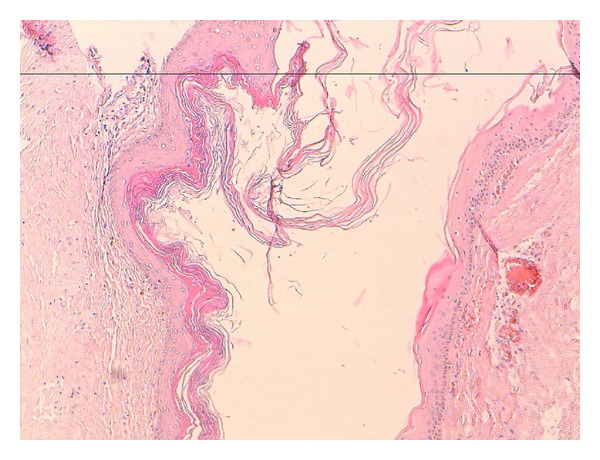
HES × 10: Microscopic image of giant epidermoid cyst with stratified squamous epithelium containing necrotic debris.

The histology was compatible with an epidermoid cyst (Figure 4). 

 The postoperative course was uneventful and the patient was doing well enough after two years of followup. 

## 3. Discussion

Epidermoid cyst is a frequent benign cutaneous tumor. It can occur anywhere on the body, and the most frequent sites involved are the face, the scalp, the neck, and the trunk [6].

Only a few cases (fifteen cases) were reported in the pelvic whose location is retroperitoneal, retrorectal, presacral [11–16], one case was reported in the round ligament [17].

 Epidermoid cysts are usually small; however, sometimes they can reach significant volumes, displacing or damaging nearby organs [2].

The pathogenesis is still unclear: congenital or posttraumatic theories are discussed [10].

Because of the rarity of epidermoid cyst, this diagnosis is rarely evoked, and is difficult in the preoperation stage. Clinical examination including pelvic examination revealed a well-limited cyst. Ultrasound and computed tomography (CT) often lead to ovarian cyst diagnosis; CT identifies better epidermoid cysts which are characterized by the absence of homogeneous fluid density, which can easily remove lipomas, fibromas, and desmoid tumors that are differential diagnosis with epidermoid cysts. 

Magnectic resonance imaging (MRI) is more specific: epidermoid cyst appears generally as hypointense on T1-weighted RM imaging, and hyperintense on T2-weighted imaging [18].

The treatment of the pelvic epidermoid cyst is a surgical ablation: the discovery of a cyst during surgery should prompt us to search for the organ on which it depends. The macroscopic appearance can eliminate a hydatid cyst. Because of the rarity of developmental cysts, they are very frequently misdiagnosed and so inappropriate surgery ensues. If a gynecologist initially finds a retrorectal cyst, most cases will be misdiagnosed as an ovarian tumor. Retrorectal epidermoid cyst contains fatty elements such as desquamated debris, cholesterol, keratin, and water. A gynecologist confuses these elements in the epidermoid cyst with those of mature cystic teratoma, which is a common ovarian tumor. However, if we have knowledge of the developmental cysts, by careful digital examination and image diagnosis, it would be possible to make a differential diagnosis since developmental cysts exist between the presacral and retrorectal space, not in the Douglas pouch like an ovarian tumor [15]. 

When there is a high suspicion of giant pelvic cyst, dissection must be done in a cleavage plane between pseudocapsule of the cyst and adjacent structures. If this dissection becomes dangerous to adjacent organs including rectum, it would be wiser to open the pseudocapsule, drain the cyst, and proceed to resection of the hull if it involves no risks what so ever for adjacent organs. The residual cavity is drained by a Redon to remove a possible hematoma collected at this level. The post-operative course is usually simple [1]. 

A successful laparoscopic excision of a retrorectal epidermoid cyst was described [15, 19].

 In the literature, no case of recurrence has been reported in followed up patients operated for a pelvic epidermoid cyst with a period ranging between 5 [10] to 26 months [19]. 

## 4. Conclusion 

Epidermoid cyst is a frequent benign cutaneous tumor. The pelvic location of this entity is rare and difficult to diagnose preoperatively, and so should be considered in the differential diagnosis of ovarian tumors. The treatment consists of a surgical ablation using a cleavage to avoid any damage for other organs.
